# Editorial: Lactate as a Major Signaling Molecule for Homeostasis

**DOI:** 10.3389/fphys.2022.910567

**Published:** 2022-06-08

**Authors:** Luc Pellerin, Philippe Connes, Catherine Bisbal, Karen Lambert

**Affiliations:** ^1^ IRMETIST Inserm U1313, Université et CHU de Poitiers, Poitiers, France; ^2^ LIBM. EA7424, Vascular Biology and Red Blood Cell Team, Université Claude Bernard Lyon 1, Lyon, France; ^3^ PhyMedExp Inserm U1046-CNRS 9214, Université de Montpellier, Montpellier, France

**Keywords:** metabolism, lactate, MCT, GPR81/HCAR1, brain, muscle

L-lactate metabolism was first investigated in exercise physiology since it was considered as a waste product of glycolysis due to oxygen deficiency. Since the 1980s’, several studies showed that lactate is an essential metabolic fuel and signaling molecule (Brooks, 2020). Then, the interest for lactate has extended to a growing number of disciplines, from physiology to pathology and exercise is now viewed as a metabolic trigger for tissues adaptation.

Lactate is transported by the monocarboxylate transporters (MCTs) family which present a ubiquitous tissue distribution but lactate can additionally activate the hydroxycarboxylic acid receptor 1 (HCAR1) also known as G protein-coupled receptor 81 (GPR81). In most tissues and cell types, lactate entry is mediated by MCT1 or MCT2 and related to oxidative capacity whereas MCT4 would be mainly involved in lactate output. The basic knowledge on lactate is that it could be a metabolic substrate or exchange molecule for muscle (Brooks), or brain (Pellerin, 2010), that MCTs content vary with training (Dubouchaud et al., 2000), nutritional status (Lambert et al., 2003; Pierre et al., 2007), cellular activity or intracellular signaling (Pérez-Escuredo et al., 2016) and diseases (Py et al., 2001).

**FIGURE 1:**
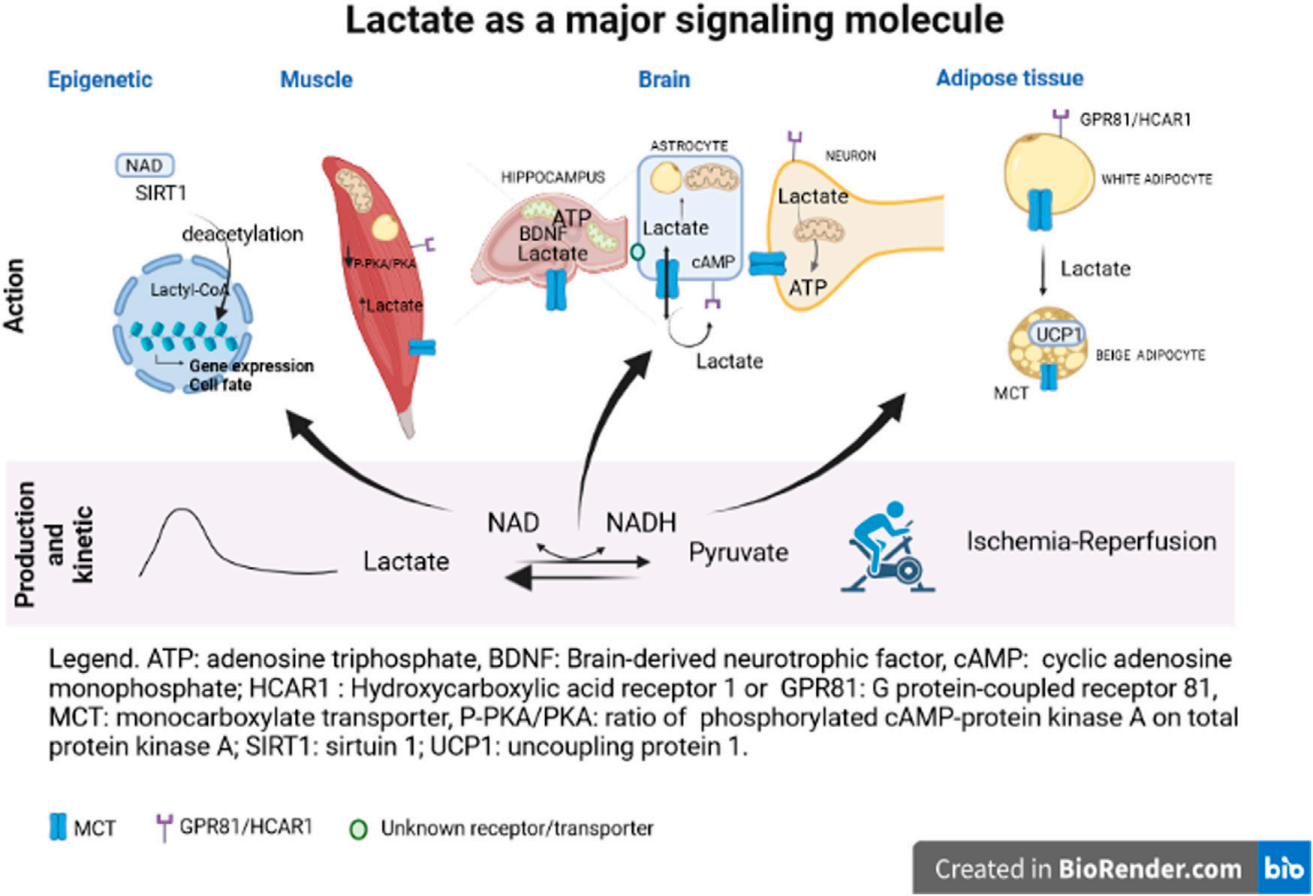
ATP, adenosine triphosphate; BDNF, brain-derived neutrophic factor; cAmp, cyclic adenosine monophosphate; HCAR1, hydroxycarboxylic acid receptor 1 or GPR81, G protein-coupled receptor 81; MCT, monocarboxylate transporter; P-PKA/PKA, ratio of phosphorylated cAMP-protein kinase A on total protein kinase A; SIRT1, sirtuin 1; UCP1, uncoupling protein 1.

This Research Topic provides an update about the knowledge of lactate roles and mechanisms of action. Durand et al., focused on blood lactate kinetics in response to exercise. Their findings showed that the longer the recovery period, the better is the quality of models to describe lactate exchange and removal abilities. In the brain, exercise allows to increase lactate release, favoring hippocampal metabolism and especially mitochondrial biogenesis (Park et al.) but also mitochondrial efficiency and brain-derived neurotrophic factor biosynthesis (Hu et al.) (Figure 1). The ability of lactate to increase mitochondrial biogenesis has been previously described in skeletal muscle. The present studies demonstrated that lactate and oxidative metabolism could play an important role in the physiology of other tissues. Indeed, the effects of exercise on hippocampal mitochondria number and function highlight the role of lactate in mediating memory processes and, in turn, physical performance. Lactate is a mediator of neuron-astrocyte dialogue since astrocytes provide lactate to neuron as an energetic substrate. Horvat et al. have provided evidence of this relationship with a focus on the lactate-positive feedback mechanism in astrocytes. Astrocytes switch their metabolism to lipid metabolism, enhancing availability of lactate for neuron as a metabolic substrate when ATP needs to be increased. The control and regulation of lactate entry into astrocytes could involve known and unknown transporters. Interestingly, during stressful conditions, such as hypoxia or ischemia-reperfusion (I/R), increasing lactate concentration *via* exogenous lactate perfusion ameliorates the neurological outcome (Roumes et al., 2021). Buscemi et al. have tested the hypothesis that the beneficial effect of lactate in cerebral I/R process could be due to the activation of HCAR1 by lactate. However, HCAR1 agonists did not exert a protective effect neither on lesion size nor on neurological outcome. A role of other transporter(s)/receptor(s) or metabolite(s) in this process is suggested.

The signaling pathway(s) activated by lactate are still unclear. Chen et al. provided interesting results suggesting the implication of the cAMP-PKA pathway. Chronic lactate injection, mimicking lactate variation during exercise, induced intramuscular triglycerides accumulation mainly through inhibition of lipolysis *via* a decrease of PKA activation. However, citrate synthase protein level and lactate concentration were still high even after PKA activation by forskolin. These findings suggest that lactate could regulate oxidative capacity through mitochondrial metabolite derivatives. This relationship could participate to the increase in performance of athletes but would be lost in muscle of obese patients. In metabolic diseases, hyperlactatemia is not associated with increased oxidative capacity, underlining a dysregulation of lactate signaling.

Adipose tissues, like skeletal muscles, have a remarkable cell and metabolic plasticity and a large ability to adapt their phenotype in response to their environment. The review of Lagarde et al. discussed the adaptability of white, brown and beige adipocytes. The authors illustrated how adipocytes are especially adapted to control the redox equilibrium at cellular and whole-body level. The adipose tissues responses to lactate exposure could be part of the tissue stress adaptation. In pathological context, an alteration of adipose tissues lactate response would disturb whole-body redox state level favoring an impairment of redox homeostasis and whole-body metabolism.

Beside its role in redox homeostasis, lactate is a strong candidate as a promoter of different cell fates such as for satellite cells and immune cells. Lactate is a regulator of redox potential *via* the NAD/NADH *ratio* leading to sirtuin deacetylases, like SIRT1, activation that will alter histones acetylation and gene expression. The epigenetic role of lactate is not solely related to its metabolic activity but also to a new process called lactylation (Zhang et al., 2019). Although there is no direct clues of satellite cell activation by lactate, the review of Nalbandian et al. discussed different experiments suggesting a role of lactate in muscle regeneration *via* activation and proliferation of satellite cells.

Several immune cell type profiles are shaped by lactate and its associated proton leading to modification of their function. As mentioned in the review of Caslin et al., immune cell metabolism has gained attention only recently. The inhibition of glycolysis in inflammatory immune cells by lactate would promote anti-inflammatory and immunosuppressive processes. However, depending on the environment, epigenetic alterations, acute or chronic inflammation, immune cells in the course of a pathological process will exhibit a different response to lactate exposure.

Although lactate has been discovered more than two centuries ago, there is still a need for research to understand lactate actions in physiology and diseases. How lactate coordinates redox metabolism at cell, tissues and whole-body level? How lactate crosses cellular membranes with a regulation at transporter/receptor levels? How lactate and glycolysis are involved in epigenetic alterations? What signaling pathways are activated by lactate? Thus, although lactate is recognized today as a cornerstone of tissues adaptation and communication, future studies are still needed to further clarify its numerous roles.

Finally, the editors would like to thank all contributors and reviewers that allowed this Research Topic to become a success in drawing attention to a long-neglected (but so interesting!) metabolite.
